# Ligamentum teres augmentation (LTA) for hiatal hernia repair after minimally invasive esophageal resection: a new use for an old structure

**DOI:** 10.1007/s00423-021-02284-9

**Published:** 2021-10-06

**Authors:** Mira Runkel, Jasmina Kuvendjiska, Goran Marjanovic, Stefan Fichtner-Feigl, Markus K. Diener

**Affiliations:** Department of General and Visceral Surgery, Medical Center, University of Freiburg, Hugstetterstrasse 55, 79106 Freiburg, Germany

**Keywords:** Minimally invasive esophageal resection, Hiatal hernia, Hiatoplasty, Ligamentum teres

## Abstract

**Purpose:**

Hiatal hernias with intrathoracic migration of the intestines are serious complications after minimally invasive esophageal resection with gastric sleeve conduit. High recurrence rates have been reported for standard suture hiatoplasties. Additional mesh reinforcement is not generally recommended due to the serious risk of endangering the gastric sleeve. We propose a safe, simple, and effective method to close the hiatal defect with the ligamentum teres.

**Methods:**

After laparoscopic repositioning the migrated intestines, the ligamentum teres is dissected from the ligamentum falciforme and the anterior abdominal wall. It is then positioned behind the left lobe of the liver and swung toward the hiatal orifice. Across the anterior aspect of the hiatal defect it is semi-circularly fixated with non-absorbable sutures. Care should be taken not to endanger the blood supply of the gastric sleeve.

**Results:**

We have used this technique for a total of 6 patients with hiatal hernias after hybrid minimally invasive esophageal resection in the elective (*n* = 4) and emergency setting (*n* = 2). No intraoperative or postoperative complications have been observed. No recurrence has been reported for 3 patients after 3 months.

**Conclusion:**

Primary suture hiatoplasties for hiatal hernias after minimally invasive esophageal resection can be technically challenging, and high postoperative recurrence rates are reported. An alternative, safe method is needed to close the hiatal defect. Our promising preliminary experience should stimulate further studies regarding the durability and efficacy of using the ligamentum teres hepatis to cover the hiatal defect.

**Supplementary Information:**

The online version contains supplementary material available at 10.1007/s00423-021-02284-9.

## Introduction

**Fig. 1:**
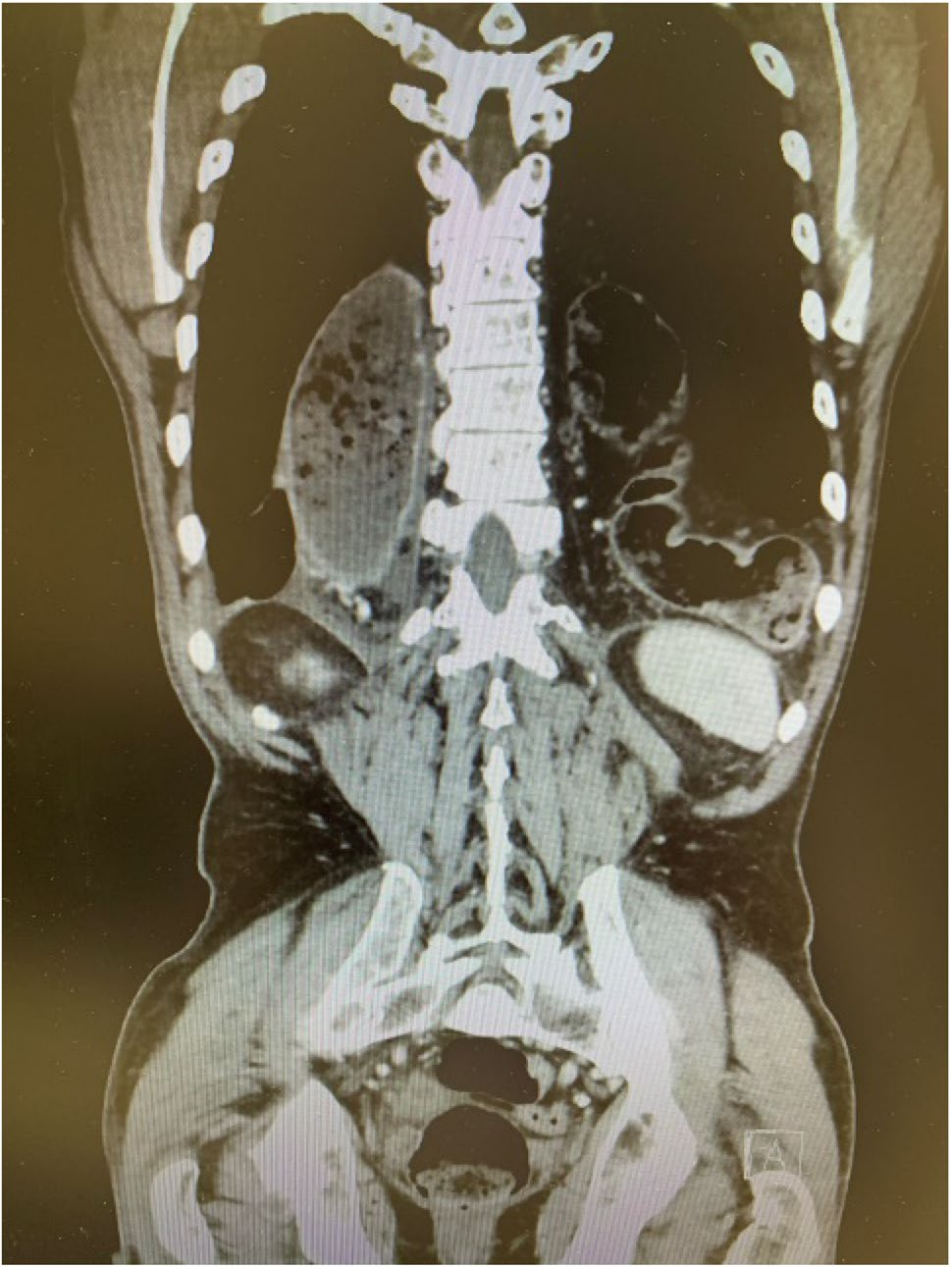
Computer tomography of a patient with a large HH with bowel contents after HMIE

Minimally invasive esophageal resection (MIE) has become the gold-standard technique for patients with malignant disease of the esophagus and the gastroesophageal junction, showing generally favourable results compared to the open approach [1–3]. Hiatal hernias (HH) after MIE are serious complications with an incidence of up to 10.2% [4–6] (Fig. 1). HH occur most likely due to the extensive widening of the hiatus to allow the gastric conduit to pass freely into the thorax. Due to the laparoscopic approach, there are less postoperative adhesions continuing to allow a degree of mobility of the gastric conduit. Furthermore, observations show an additional widening of the hiatal orifice postoperatively, possibly due to breathing mechanics and initial contraction. Only few case reports and limited retrospective studies have analyzed the diagnosis, treatment and complications for HH after MIE [4, 5, 7–9]. Price et al. analyzed over 2000 patients from a single instutition, where hiatoplasty with or without mesh reinforcement was the surgical treatment option for HH after MIE. Morbidity rates up to 60% and recurence rates of 13.3% were reported [4]. A smaller cohort analysed by Kent et al. showed morbidty rates of 27% and recurrence rates up to 29%, with or without the use of mesh [5]. High recurrence rates may be due to the wide defects and often scarred and unflexible crura, making an approximation of the hiatus technically challenging. Due to the risk of serious complications, the use of mesh is highly controversial and generally not even recommended for the closure of primary hiatal hernias [10–12]. Thus, there is a need for an alternative, safe and more durable technique to cover the hiatal defect. We developed an innovative method of covering the post-MIE hiatal defect by using a flap of the ligamentum teres hepatis.

The ligamentum teres hepatis (also known as the round ligament of the liver) is the remnant of the obliterated umbilical vein. It is the free, inferior edge of the falciforme ligament which spans between the liver and the diaphragm and anterior abdominal wall. The blood supply arises from the left phrenic artery and a ligamental branch of the middle hepatic artery which allows the construction of a vascularized flap pediculated at the liver [13]. This paper is a step-by-step description of our uncomplicated technique of the laparoscopic ligamentum teres augmentation (LTA) for patients after esophageal resections.

## Methods and operative technique

**Fig. 2:**
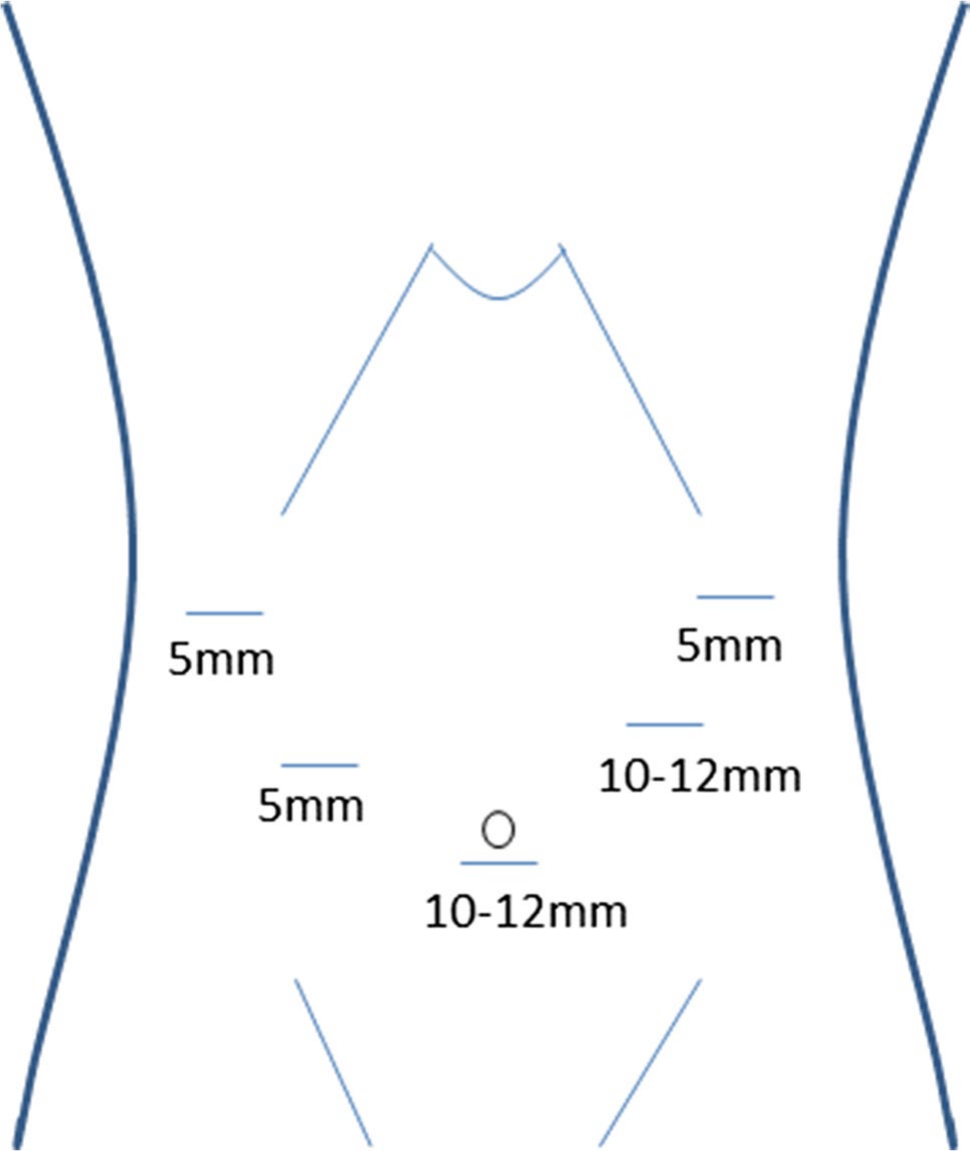
Sketch of the trocar placement for ligamentum teres augmentation (LTA)

**Fig. 3:**
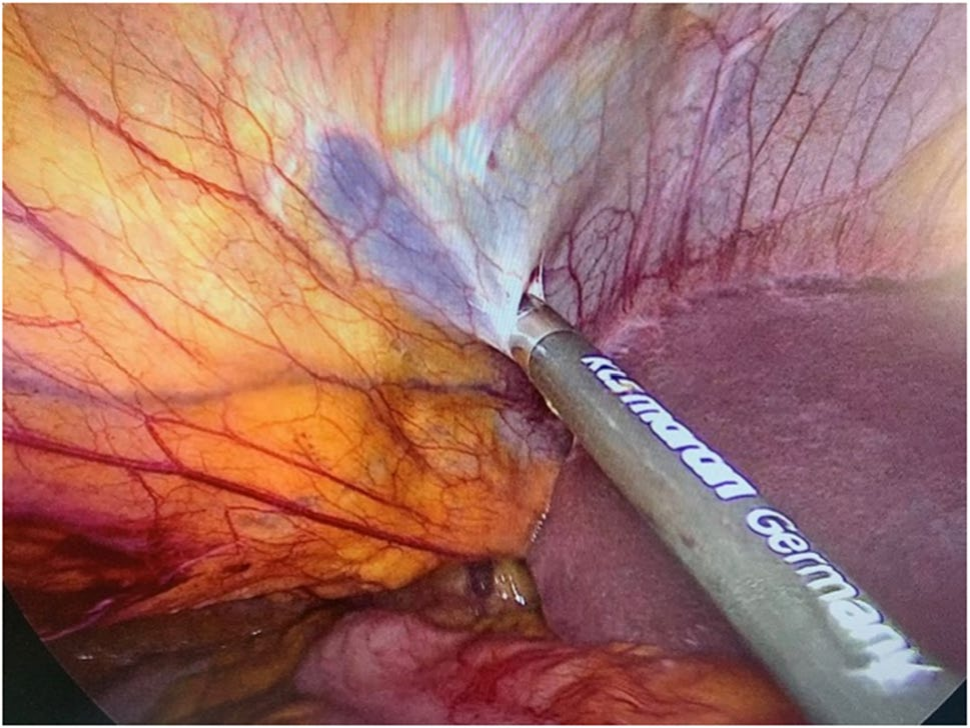
Dissection of the ligamentum teres hepatis from the ligamentum falciforme and the anterior abdominal wall using a harmonic device. The vascular supply from the liver through the ligamental artery is preserved. Laparoscopic view from the left

**Fig. 4:**
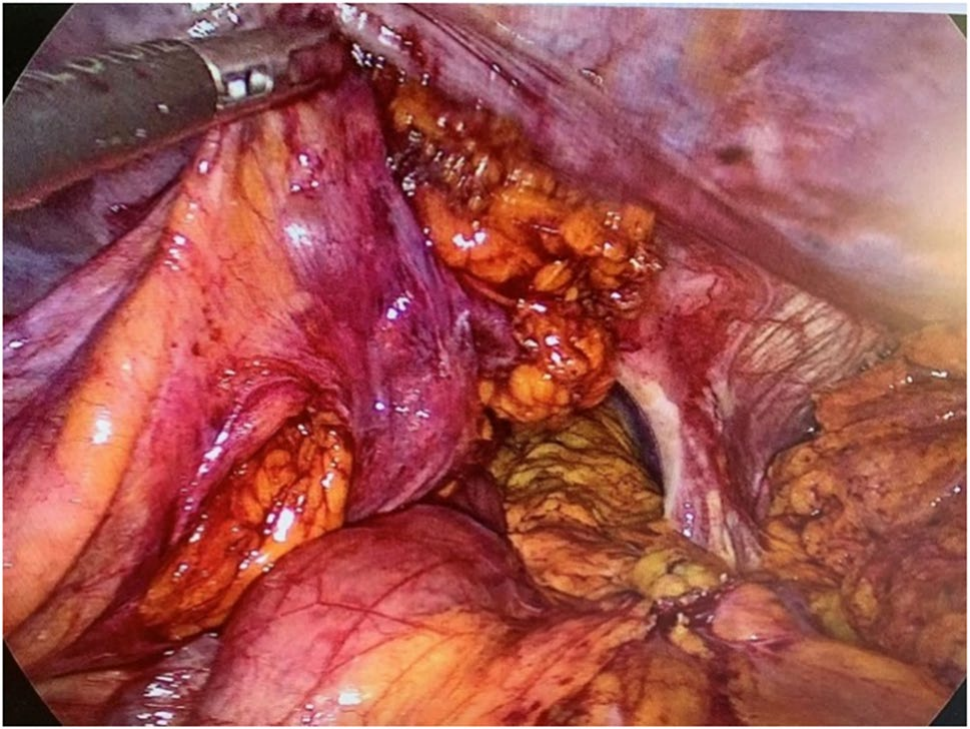
The ligamentum teres flap is swung behind left liver lobe toward the hiatus. Laparoscopic view onto the wide hiatal defect around the gastric conduit

**Fig. 5:**
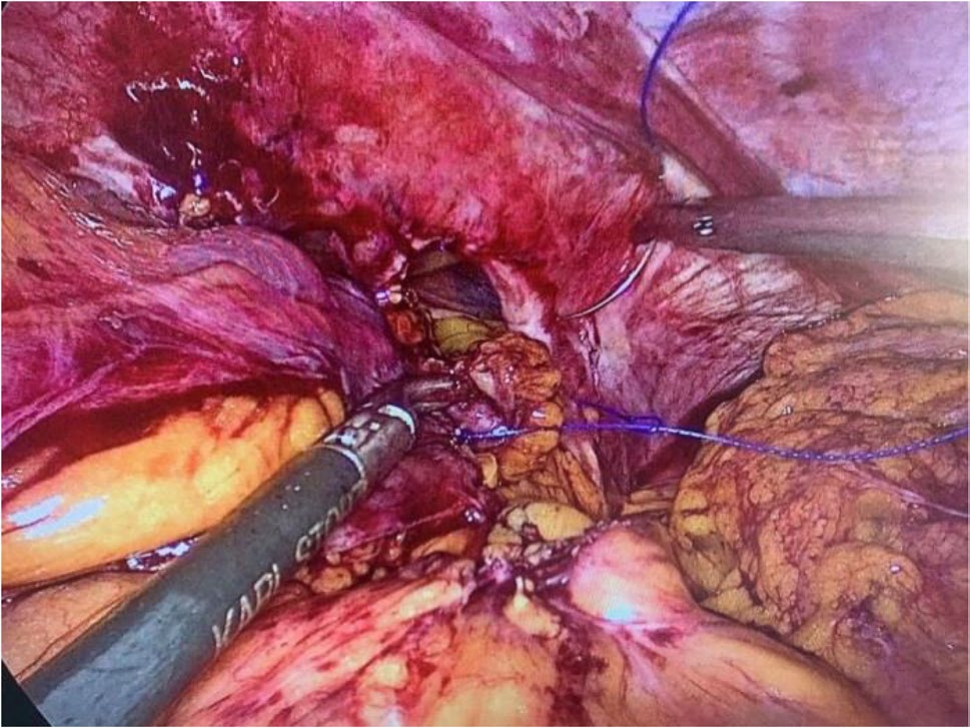
Semi**-**circular fixation of the ligamentum teres flap along the anterior hiatal margin. Laparoscopic view toward the hiatus, with sutures starting at the left crura

**Fig. 6:**
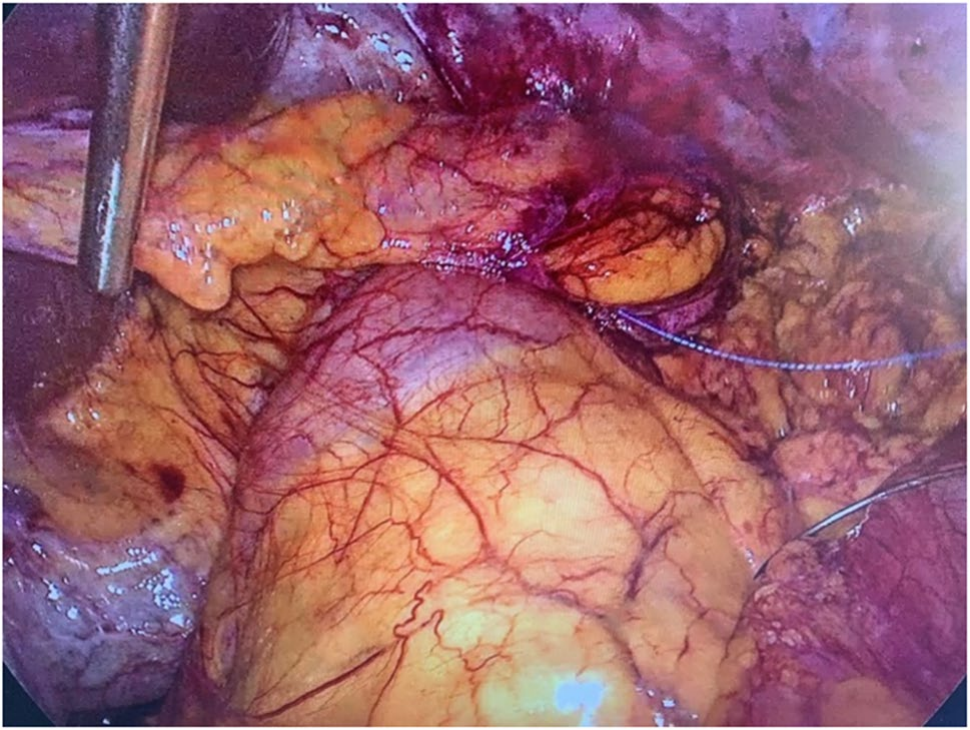
Fixation of the ligamentum teres flap to the ventral wall of the gastric conduit. Laparoscopic view toward the hiatus

**Fig. 7:**
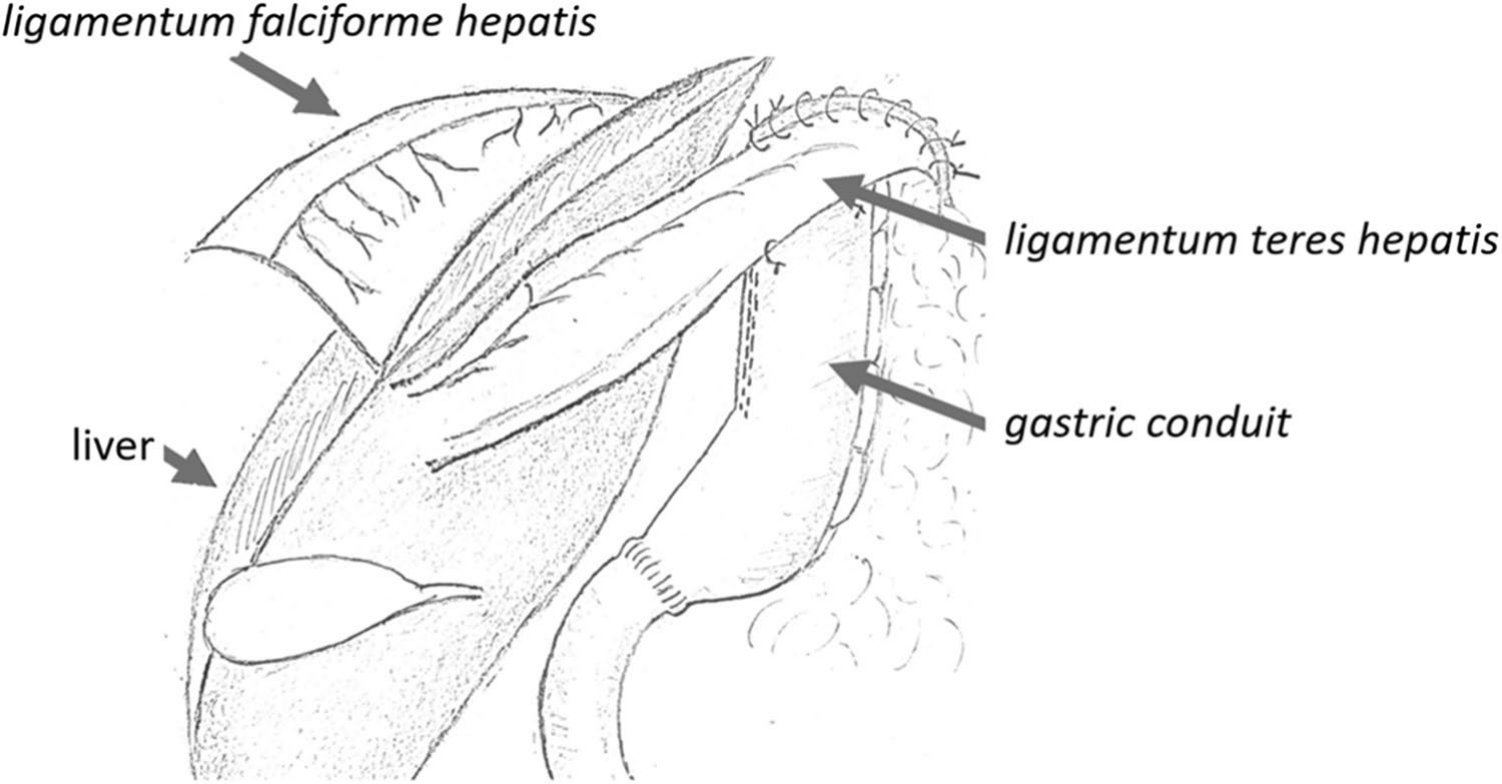
Final outcome after ligamentum teres augmentation after esophageal resection with gastric conduit

Laparoscopy is performed via five trocars with the patients in standard French position (Fig. 2). First, adhesiolysis and dissection of the hiatal opening is carried out until both crura are visualized. The gastric conduit is identified, since its blood supply at the greater curvature must be secured. Then the migrated abdominal contents are repositioned into the abdominal cavity. Second, the ligamentum teres is dissected from the ligamentum falciforme and the anterior abdominal wall using an energy device (Fig. 3). Third, the mobilized and pediculated ligamentum teres hepatis is positioned behind the left liver lobe and swung up to the left towards the hiatal orifice (Fig. 4). Fourth, the ligamentum is placed anterior to the gastric conduit and anchored to the left crura with non-absorbable sutures. Once spread out and flattened, it is then semi-circularly sutured along the anterior hiatal margin with final fixation to the right crura (Fig. 5). Care is taken as not to puncture the pericardium. Fifth, the ligamentum is finally anchored to the ventral aspect of the gastric conduit with special attention not to endanger the blood supply of the gastric conduit via the right gastroepiploic artery (Figs. 6 and 7, supplementary video). Finally, the trocars are removed and the wounds closed. Drains are usually not required.

## Results

We have used the LTA for primary (*n* = 4) and revisional repairs (*n* = 2) of post-hybrid MIE hiatal hernias, both in the elective (*n* = 4), as well as emergency setting (*n* = 2). No technical difficulties or intraoperative complications were encountered. Operating time ranged from 79 to 110 min in the elective setting and 125–160 min in the emergency setting. One patient experienced a complication due to anastomotic leak after small bowel resection, not related to the LTA, and underwent revisional surgery. All patients were discharged in a timely manner between 5 and 6 days, except for the patient with the anastomotic leak (49 days). In postoperative follow-up, one patient reported occasional vomiting, which subsided after stimulation, and one patient reported mild dysphagia. No abnormalities could be detected on gastroscopy. We have not observed any clinical or radiological recurrence within the short-term follow-up of 3 months in 3 cases.

## Discussion

Hiatal hernias are a potentially life-threatening complication after minimally invasive esophageal resection (MIE). The technical difficulty and high recurrence rates after suture-hiatoplasty and the risk associated with mesh-repair have led us to consider the use of the ligamentum teres hepatis for an alternative closure of the hiatal defect. The “old” ligamentum teres has been revived during recent years for a variety of surgical procedures, including reconstruction after perforated duodenal ulcers, covering of a pancreatic stump after pancreas resections and various vessel and bile duct reconstructions [14]. Promising short-term results have been published for the ligamental repair of small primary hiatal hernias; however, recurrence rates were as high as 60% for large hiatal defects (> 9 cm) [15, 16]. In the bariatric population, the use of the ligamentum teres has been described for intrathoracic migration after one-anastomosis gastric bypass and sleeve gastrectomy with promising results [17]. The purpose of the ligamental flap is the anchoring of the sleeve of pouch into the abdomen and therefore, it is placed posteriorly around the gastroesophageal junction as a 270–360˚ sling.

In our technique of the LTA, the flap acts as a cover for the hiatal opening and is placed anteriorly as not to endanger the blood supply to the gastric conduit. In our experience, this technique addresses the specifics of the postoperative problem of HH after MIE due to several reasons:

The ligamentum teres provides highly flexible and tension-free coverage of the hiatus. Wide hiatal defects with unflexible, scarred cruras can be covered sufficiently, while still allowing movement for breathing.

Due to the use of biologic, autologous material to cover the hiatus, the risks compared to using synthetic mesh (e.g. erosions) are eliminated.

The ventral fixation to the gastric conduit provides stability, while still allowing some movement and dilatation of the gastric conduit during digestion.

A similar method of reconstruction using a falciform ligament flap for hiatal closure after and during esophagectomy has recently been described by Asti et al., who used ICG-green to assess vascularization before and after mobilisation [18]. In this small retrospective analysis, the falciform ligament was used to buttress a suture hiatoplasty, with promising results both in anatomical recurrence and symptomatic control. Although the two techniques differ slightly, it can be assumed that a reconstruction of the hiatal orifice using the ligamentum teres or falciforme is a viable and safe alternative treatment option for HH repair after esophagectomy and warrants further research. The use of ICG-green could also enhance reconstruction techniques, although in our experience, no issues regarding vascularization of the flap were encountered. Despite promising results in few case series, the long-term results from prospective studies must be awaited before superiority can be established and the LTA can be recommended as a standard method of reconstruction. Until then, primary suture repair hiatoplasty remains a valid option for HH repair after MIE.

Additionally, simultaneous HH repair during MIE has been described. Although potentially decreasing the incidence of postoperative HH, the influence of postoperative oedema and potential overtreatment are unknown. Furthermore, the LTA provides technical challenges during simultaneous esophageal reconstruction, due to the patients’ position and flexibility of the gastric sleeve to be pulled up into the thorax.

Although the proposed technique has shown good clinical outcomes with no additional morbidity, other alternative or supplementary procedures, e.g., colopexy, could be considered to further improve recurrence rates [19, 20].

## Conclusion

Our promising preliminary experience with this minimally invasive procedure of low complexity using the ligamentum teres to cover the hiatal defect without tension after esophageal resection should stimulate further exploration of this new alternative and test its efficacy and durability.

## Supplementary Information

Below is the link to the electronic supplementary material.

ESM 1 (MP4 645 mb)

## Data Availability

All data and material are available at request. Please contact corresponding author.
